# The effects of resistance exercise with blood flow restriction on muscle performance, muscle mass, and function in older adults: a systematic review

**DOI:** 10.1186/s11556-026-00425-x

**Published:** 2026-07-10

**Authors:** Matic Jelen, Manca Opara Zupančič, Nejc Šarabon

**Affiliations:** 1 Department of Physiotherapy, Faculty of Health Sciences, University of Ljubljana, Ljubljana, Slovenia; 2 Faculty of Health Sciences, University of Primorska, Koper, Slovenia; 3 Ludwig Boltzmann Institute for Rehabilitation Research, Vienna, Austria

**Keywords:** Aging, Strength, Power, Endurance, Frailty, Hypertrophy, BFR, Ischemia

## Abstract

**Background:**

Blood flow restriction resistance exercise is emerging as a promising strategy for older adults who are unable to perform high intensity resistance exercise. The purpose of this systematic review was to evaluate the effects of blood flow restriction resistance exercise on muscle performance, muscle mass, and function in healthy older adults.

**Methods:**

A systematic literature review was conducted in the PubMed, Cochrane Central, PEDro, and ScienceDirect databases. Randomized controlled trials published in English up to May 2026 were included. Eligible studies involved healthy older adults (mean age ≥ 60 years) and compared blood flow restriction resistance exercise with low intensity resistance exercise, high intensity resistance exercise, or non-exercise control group. Six studies were included in the analysis, comprising 197 participants. Due to heterogeneity of outcome measures and intervention protocols, results were synthesized descriptively. The methodological quality of the included studies was assessed using the PEDro scale, and the risk of bias was evaluated using the Cochrane Risk of Bias tool for randomized controlled trials (RoB 2.0).

**Results:**

Blood flow restriction resistance exercise may improve lower-limb muscle strength and cross-sectional area, particularly of the *quadriceps femoris* muscle, with effects comparable to traditional high intensity resistance exercise. One-repetition maximum increased by 15.6–54%, and *quadriceps femoris* cross-sectional area increased by 3.23–4.2 cm². Limited data were available for functional outcomes but suggested potential benefits. No adverse side effects were reported. None of the included studies was judged to be at overall low risk of bias.

**Conclusions:**

Despite methodological limitations, small sample sizes, and heterogeneity of exercise protocols, blood flow restriction resistance exercise appears to be a safe and effective alternative for improving lower-limb muscle strength and muscle mass in healthy older adults. Certainty of evidence is limited by risk of bias and the small number of available studies. Future research should investigate long-term and upper-limb effects and standardized exercise protocols to support the development of clinical guidelines. This research was financially supported by the Slovenian Research and Innovation Agency (KINSPO, P5-0443; grant No. 58589), and the review was not prospectively registered.

## Background

Aging is associated with a decline in muscle performance, muscle mass, and function [1], which may lead to reduced physical capacity [2], decreased independence [3], an increased risk of frailty [4], falls [5], the development of chronic diseases, and higher mortality [6]. One of the most effective methods for preventing these complications and for maintaining muscle performance, muscle mass, and function in older adults is resistance exercise (RE) performed at loads of 70–85% of one-repetitin maximum (1RM) [7, 8]. However, many older adults are often unable to perform high intensity RE due to limitations such as pain and comorbidities [9]. Although BFRE may therefore be clinically relevant for these populations, this type of exercise may also be beneficial for otherwise healthy older adults who experience psychological or functional barriers, such as fear of falling or injury, pronounced muscle weakness, or reduced mobility.

In recent years, increasing attention has therefore been directed toward low intensity RE with blood flow restriction (BFR) [10]. Blood flow restriction exercise (BFRE) is an exercise method in which an inflatable cuff is placed on the proximal portion of the upper or lower limbs that partially restricts arterial inflow and completely occludes venous outflow from the active muscles during physical activity [11, 12]. This mechanically induced occlusion leads to a hypoxic state, characterized by reduced oxygen and nutrient delivery to the tissue [13]. The advantage of BFR-RE is the use of substantially lower loads (20–40% 1RM) compare with traditional RE, resulting in lower mechanical stress on the body, which is particularly important for individuals who are unable to perform high intensity RE [11, 14].

Previous systematic reviews have reported positive effects of BFRE across different populations and exercise modalities. For example, BFRE has been associated with improvements in muscle strength and muscle mass in older adults [7], strength in musculoskeletal rehabilitation [10], muscle strength and size after anterior cruciate ligament reconstruction [15], pain and strength in knee osteoarthritis [16], and strength, power, speed, endurance, and body composition in athletes [17]. Beneficial effects have also been reported in heart disease and heart failure, including improvements in skeletal muscle strength, endurance, exercise tolerance, and functional performance [18], while reviews in sarcopenia suggest positive effects of resistance exercise and potential benefits of BFRE for muscle strength and physical performance [19, 20]. In neurological disorders, BFRE has been associated with improvements in gait speed, walking endurance, fatigue, quality of life, and muscle thickness, although evidence remains limited and heterogeneous [21]. However, these reviews included heterogeneous BFR modalities, such as BFR during resistance exercise, walking, aerobic exercise, and rehabilitation-based protocols, as well as populations that did not specifically represent healthy older adults.

Based on a review of existing systematic reviews and meta-analyses, we found that this systematic review differs primarily by including a homogeneous population of healthy older adults, focusing exclusively on RE with BFR, and providing a comprehensive assessment of muscle performance, muscle mass, and physical function. Existing reviews frequently include heterogeneous populations or various forms of BFRE, which limits the direct applicability of their findings to clinical practice in healthy older adults.

Consequently, there is currently no systematic review that comprehensively summarizes the effects of BFR-RE on muscle performance, muscle mass, and function in healthy older adults.

The aim of this systematic review was therefore to examine the effects of BFR-RE on muscle performance, muscle mass, and function in older adults and to provide guidelines for clinical practice. We hypothesized that BFR-RE improves muscle performance, muscle mass, and function in this population.

## Methods

### Inclusion and exclusion criteria

The inclusion and exclusion criteria were defined in accordance with the PICOS framework [22]:

- (P) Population: Studies were included if they involved healthy older adults of male and/or female sex, with a mean sample age ≥ 60.0 years. Studies that included participants with previous surgical procedures or with known diseases, such as rheumatoid arthritis, sarcopenia, heart failure, stroke, multiple sclerosis, obesity, osteoporosis, osteoarthritis, osteoarthrosis, chronic pain, diabetes mellitus, hypertension, dementia, Parkinson’s disease, and implanted endoprostheses, were excluded. The restriction to healthy older adults was applied to reduce clinical heterogeneity and to allow a clearer interpretation of the effects of BFRE in older adults without diagnosed chronic diseases or major health conditions.
- (I) Intervention: Studies were included if they examined BFRE, regardless of study duration. Studies in which BFR-RE was combined with other approaches, such as hydrotherapy, aerobic exercise, and balance training, were excluded.
- (C) Comparison: Studies were included if the comparison group did not participate in any intervention or performed low intensity or high intensity RE. Studies without a comparison group were excluded.
- (O) Outcomes: Studies were included if they assessed at least one of the following outcomes: muscle performance, muscle mass, or function, regardless of the type of measurement tool or body region. Studies that did not assess any of these outcomes were excluded.
- (S) Study design: Studies were included if they were randomized controlled trials with at least one BFR-RE intervention group and one comparison group. Non-randomized studies, single-group studies, reviews, and other non-original research articles were excluded.

### Search strategy

The systematic review was conducted in the databases PubMed, Cochrane Central Register of Controlled Trials, PEDro, and ScienceDirect. These databases were selected to cover biomedical literature, randomized controlled trials, physiotherapy-specific evidence, and broad scientific literature relevant to BFR-RE in older adults. However, we acknowledge that other databases commonly used in exercise science and rehabilitation, such as Embase, SPORTDiscus, and CINAHL, were not searched, which may have reduced search sensitivity. Randomized controlled trials published in English with full-text availability up to May 2026 were included. For databases that allowed the use of Boolean operators, the following combination of keywords was applied: (“blood flow restriction” OR “BFR” OR “BFRE” OR “BFRRE” OR “KAATSU” OR “occlusion train*” OR “occlusion exercise*” OR “vascular restriction” OR “tourniquet train*” OR “vascular occlusion” OR “ischemic train*” OR “ischemic exercise*”) AND (“older adult*” OR “aging” OR “senior*” OR “geriatric” OR “elder*” OR “old age” OR “aged” OR “age-related” OR “old people” OR “senior”). In other databases, the keyword combinations were adapted accordingly. The search was conducted in three phases: (1) assessment of study relevance based on the title, (2) assessment of study relevance based on the abstract, and (3) assessment of study relevance based on the full text. All retrieved studies were imported into Mendeley (Version 2.131.0), and duplicate records were removed. Study selection was conducted using a verification approach. One reviewer performed the initial screening and selection, and all decisions were subsequently checked by two additional reviewers. Any uncertainties or disagreements were resolved through discussion until consensus was reached. Any uncertainties were resolved through discussion until consensus was reached. The review was conducted in accordance with the PRISMA 2020 guidelines and was not prospectively registered. Registration was not completed because the review was initiated as part of an internal research project before protocol registration was considered; however, the eligibility criteria, search strategy, outcomes, and methods of synthesis were defined before full-text screening and data extraction.

### Data extraction

Data from the included studies were extracted to obtain the following information: (1) study characteristics (authors, year, title, and design), (2) participant characteristics (sex and age), (3) intervention characteristics (exercise selection, load determination, number of repetitions, duration of muscle contraction, number of sets, rest duration between sets and exercises, number of exercise sessions, determination of cuff pressure, cuff width, and time under occlusion), and (4) main outcomes and measurement instruments used. Data extraction was conducted using a verification approach. One reviewer extracted the data, and all extracted data were subsequently checked by two additional reviewers for accuracy. Any discrepancies were resolved through discussion until consensus was reached. The extracted data were entered and managed in Microsoft Excel for Microsoft 365 MSO (Version 2502, Build 16.0.18526.20144, 64-bit).

Due to heterogeneity of outcome measures, results were summarized descriptively using reported mean changes and percentage improvements.

### Quality assessment of studies

Methodological quality and risk of bias assessments were conducted using a verification approach. One reviewer performed the initial assessments, which were subsequently checked by two additional reviewers. Any disagreements were resolved through discussion until consensus was reached.

The methodological quality of the included studies was assessed using the PEDro scale, which is specifically designed to evaluate randomized controlled trials in physiotherapy. The PEDro scale has been shown to be a valid tool for assessing the methodological quality of clinical trials [23]. It comprises ten criteria that allow for a systematic evaluation of study quality. Studies scoring 9–10 points were classified as “excellent,” those scoring 6–8 points as “good,” scores of 4–5 indicated “acceptable” quality, and studies scoring fewer than 4 points were classified as “low” quality [24].

The risk of bias in the included studies was assessed using the Cochrane Risk of Bias tool for randomized controlled trials (RoB 2.0). Due to the small number of studies and heterogeneity of outcomes, a formal assessment of reporting bias across studies (e.g., funnel plots or statistical tests) was not conducted. The RoB 2.0 covers five domains: the randomization process, deviations from intended interventions, missing outcome data, measurement of outcomes, and selection of reported results. A study was considered to have a low risk of bias if it was rated as low risk in all five domains or had some concerns in at most one domain. If some concerns were identified in at least two domains, the study was classified as having a moderate risk of bias. The presence of a high risk of bias in any domain classified the study as having a high risk of bias [25].

### Results of the review

**Fig. 1:**
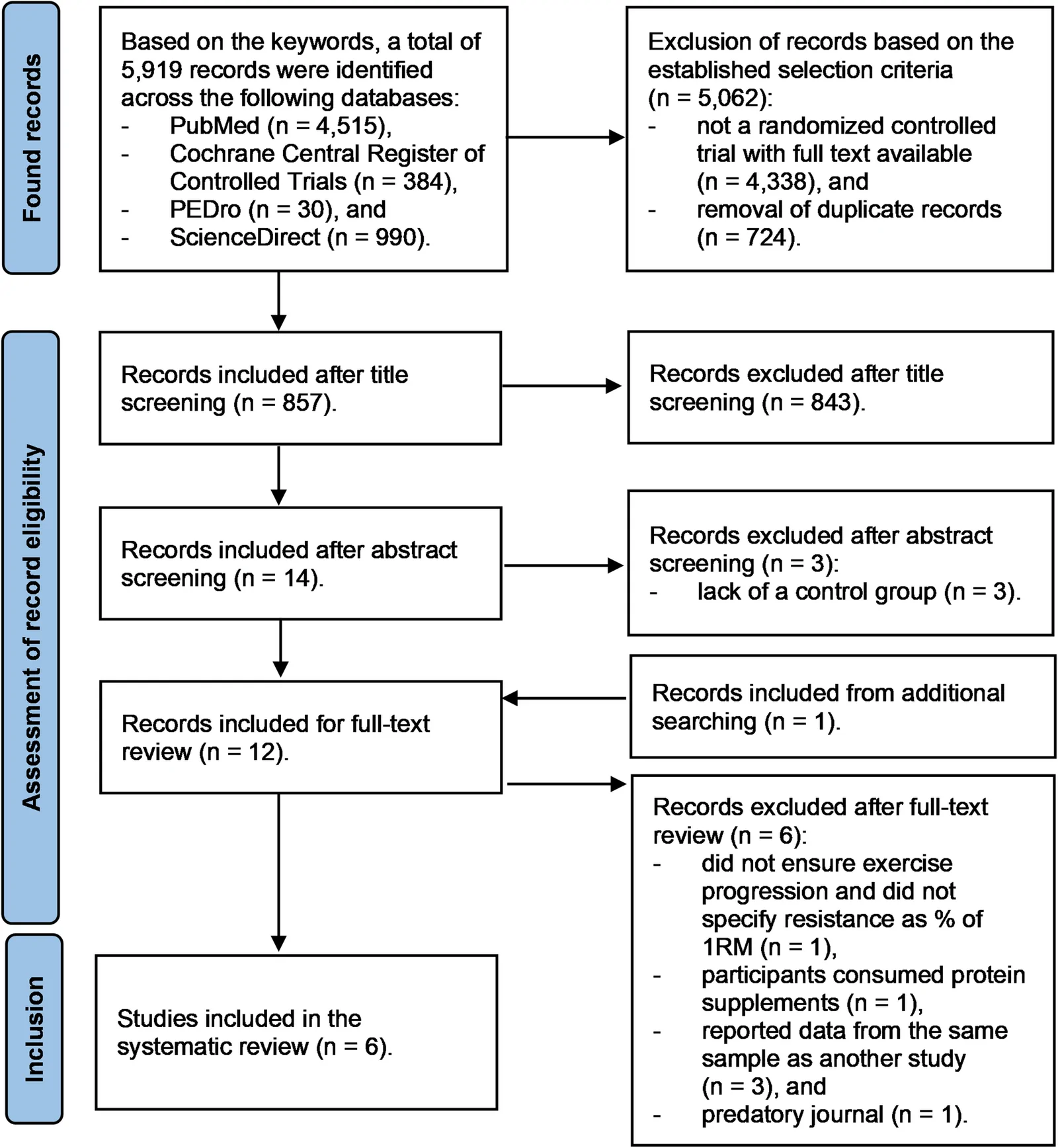
PRISMA flow diagram of the study search and selection process [26]

We identified a total of 6,190 records. After removing duplicates using Mendeley (Version 2.131.0), 861 records were included for further screening. Following title screening, 14 records remained, and after abstract screening, 11 records were retained. Additionally, one study was identified through screening the reference lists of existing systematic reviews and meta-analyses. After full-text assessment, six studies were included in the systematic review. The study selection process is presented in Fig. 1 using a PRISMA (Preferred Reporting Items for Systematic Reviews and Meta-Analyses) flow diagram [26].

## Results

### Methodological quality

PEDro scale ratings of the included studies ranged from 4 to 6, indicating acceptable to good methodological quality, with a mean of 5.17 (standard deviation = 0.75), a median of 5, and an interquartile range of 5–6. No study was rated as low or excellent quality. None of the included studies met the following criteria: (2) concealed allocation, (4) blinding of subjects, (5) blinding of therapists, and (8) intention-to-treat analysis. Scores for individual PEDro scale criteria for each included study are presented in Table 1.

**Table 1:** Assessment of the methodological quality of the included studies using the PEDro scale

AUTHOR (YEAR)	*R*. A.	C. A.	B. C.	BOS	BOT	BOA	AF-U	ITTA	BGSC	PEAV	TOTAL	QUALITY
Cook et al. (2017) [27]	✔	✘	✔	✘	✘	✔	✔	✘	✔	✔	6/10	good
Letieri et al. (2018) [28]	✔	✘	✔	✘	✘	✔	✔	✘	✔	✔	6/10	good
Shimizu et al. (2016) [29]	✔	✘	✔	✘	✘	✘	✔	✘	✔	✔	5/10	acceptable
Vechin et al. (2015) [30]	✔	✘	✔	✘	✘	✘	✘	✘	✔	✔	4/10	acceptable
Wang et al. (2023) [31]	✔	✘	✔	✘	✘	✘	✔	✘	✔	✔	5/10	acceptable
Yasuda et al. (2014) [32]	✔	✘	✔	✘	✘	✘	✔	✘	✔	✔	5/10	acceptable

*AF-U* adequate follow-up, *B. C.* baseline comparability, *BGSC *between-group statistical comparisons, *BOA *blinding of assessors, *BOS *blinding of subjects, *BOT *blinding of therapists, *C. A.* concealed allocation, *ITTA *intention-to-treat analysis, *PEAV *point estimates and variability, *R. A.* random allocation, ✔ = criterion met; ✘= criterion not met

All six studies had at least two domains rated as presenting an unclear risk of bias according to the RoB 2.0 tool, with a mean of 2.83 such domains per study (SD = 0.75), a median of 3, and an interquartile range of 2–3. Each study had one domain rated as high risk of bias; consequently, none of the studies was judged to be at overall low risk of bias. Notably, all studies were rated as high risk of bias in the second RoB 2.0 domain (bias due to deviations from intended interventions), while an unclear risk of bias was consistently observed in the first domain (bias arising from the randomization process) and the fifth domain (bias in selection of the reported result). Detailed domain-level ratings are presented in Table 2.

**Table 2:** Assessment of risk of bias in the included studies using RoB 2.0

AUTHOR (YEAR)	Rando	DFII	Missing OD	MOO	SORO	QUALITY
Cook et al. (2017) [27]	?	-	+	+	?	-
Letieri et al. (2018) [28]	?	-	+	+	?	-
Shimizu et al. (2016) [29]	?	-	+	?	?	-
Vechin et al. (2015) [30]	?	-	?	?	?	-
Wang et al. (2023) [31]	?	-	?	+	?	-
Yasuda et al. (2014) [32]	?	-	+	?	?	-

*DFII *deviations from intended interventions, *MOO *measurement of outcomes, *OD *outcome data, *rando *randomization, *SORO *selection of reported outcomes; + = low risk of bias; ? = unclear risk of bias; - = high risk of bias

### The effects of resistance exercise with blood flow restriction on muscle performance, muscle mass, and function in older adults

Characteristics of the included studies, samples, interventions, occlusion parameters, measurement instruments, and main findings are presented in Table 3 (Appendix). To improve interpretability, effect magnitudes are reported throughout the Results section as absolute and/or relative mean changes when these data were available from the included studies; where sufficient data were not reported, additional effect magnitudes were not calculated to avoid introducing unsupported estimates.

The six included studies were published between 2014 [32] and 2023 [31] and included a total of 197 participants. Intervention duration ranged from four to 16 weeks, with one study lasting four weeks [29], one study six weeks [31], three studies 12 weeks [27, 30, 32], and one study 16 weeks [28]; exercise frequency varying from two [27, 30, 32] to three sessions per week [28, 29, 31]. Most studies investigated lower-limb BFR-RE, including knee extension, leg press, knee flexion, and squats [27, 28, 30–32], while one study also included upper-limb exercises, namely chest press and horizontal rowing [29]. Across the included BFR-RE protocols, exercise intensity ranged from 20% to 30% of 1RM, and cuffs were applied proximally on the thigh fr lowerlimb exercises and proximally on the upper arm for upper-limb exercises. Cuff pressure prescription varied substantially between studies. Two studies used arterial occlusion pressure-based prescription, applying 50% AOP plus 5 mmHg [31] or 80% AOP [28]. Other studies used absolute pressre or blood pressure-based aproaches, including 120–270 mmHg [32], 1.5 × brachial systolic blood pressure [27], femoral artery systolic blood pressure [29], and 50% of systolic blood pressure [30]. This variability indicates considerable heterogeneity i pressure prescription and limits direct comparison of the physiological stimulus across studies. Because individualized pressure prescription based on arterial occlusion pressure is increasingly recommended to improve consistency and safety, this heterogeneity should be considered when interpreting the findings [33].

All included studies reported improvements in muscle strength following BFR-RE. Muscle strength was assessed using 1RM, 3RM, maximal voluntary isometric contraction, isokinetic torque, and endurance-based tests. For knee extension, mean increases ranged from 15.6% to 26.1% [31, 32], while absolute 1RM gains ranged from 8.9 kg to 9.1 kg [27, 29]. During horizontal leg press, mean 1RM improvements ranged from 33.4% to 54% [30, 32], crresponing to absolute gains between 15.7 kg and 99 kg [27, 29, 30]. For knee flexion, one study reported a mean 1RM increase of 8.2 kg [27]. Sample sizes in the BFR-RE groups were small, ranging from 8 to 20 participants, and these results should therefore be interpreted as study-level descriptive findings rather than pooled estimates.

When compared with traditional high intensity RE, no statistically significant between-group differences were generally observed despite similar improvements in studies with relatively small sample sizes. The only exception was Cook et al. [27] (*n* = 12 per group), in which the HIRE group showed significantly greater improvement in knee extension 1RM (+ 21.2 kg vs. + 9.1 kg in the BFR-RE group; *p* < 0.05).

Muscle strength was also assessed using maximal voluntary isometric contraction of the knee extensors. One study observed a mean increase of 11.5% following BFR-RE [31], whereas another reported improvement only in the traditional high intensity RE group (19.3 N⋅m); however, the between-group difference was not statistically significant [27].

Isokinetic torque of the knee flexors and extensors was additionally evaluated in one study, which reported mean post-intervention improvements ranging from 14.02 N⋅m to 25.96 N⋅m for extensors and from 15.02 N⋅m to 20.23 N⋅m for flexors following BFR-RE. A trend toward greater improvement was observed in the higher cuff-pressure group, although these changes were not statistically significant, including comparisons with traditional high intensity RE [28].

Muscle hypertrophy following BFR-RE was reported in all studies that assessed this outcome [27, 30, 32]. Mean increases in *quadriceps femoris* cross-sectional area ranged from 3.23 cm² to 4.2 cm² [27, 30], corresponding to relative gains between 7.9% and 8% [30, 32] in stdies wih BFR-RE group sample sizes ranging from 9 to 20 participants. Traditional high intensity RE showed slightly lower values, ranging from 2.86 cm² [27] to 4 cm² and approximately 6.6% increases [30]. Although betwen-group differences were not statistically significant, the results suggested a trend toward greater hypertrophy with BFR-RE. Additional increases were also reported in the hip adductors (6.5%) and *gluteus maximus* (4.4%) ollowing BFR-RE [32].

Functional and endurance-related outcomes were reported in two studies. Wang et al. [31] (*n* = 12) reported a 37% man increase in quadriceps endurance and a 7.8% icrease in 6-minute walk distance, reflecting improved submaximal functional exercise capacity. Similar findings were observed by Yasuda et al. [32] (*n* = 9), who reported an 18.3% mean ncrease in repetitions during the 30-second sit-to-stand test, reflecting improved lower-limb functional performance.

## Discussion

The aim of this systematic review was to examine the effects of BFR-RE on muscle performance, muscle mass, and function in older adults. Six randomized controlled trials were included in the analysis, each involving at least one group performing BFR-RE and at least one comparison group.

We hypothesized that BFR-RE improves muscle performance, muscle mass, and function in older adults. The results of the included studies largely support this assumption, as they reported positive effects of BFR-RE on lower-limb muscle strength, particularly of the *quadriceps femoris* muscle. In addition, individual studies reported increases in muscle mass, confirming the anabolic potential of this type of exercise in older adults. The effect of BFR-RE on function remains relatively underexplored, as only a limited number of studies have examined this domain and often did not include a comparison group performing traditional RE. Consequently, further research is required to enable a comprehensive understanding of the effects of BFR-RE on function in older adults. The main findings of our systematic review are as follows:

1. BFR-RE effectively increases 1RM in knee extension on a machine, horizontal leg press, and knee flexion on a machine, with effects comparable to traditional high intensity RE. Only one study [27] reported a greater increase in 1RM following traditional high intensity RE compared with BFR-RE. However, because a validated minimal clinically important difference for 1RM in older adults was not identified, the clinical relevance of this between-group difference remains uncertain. Therefore, conclusions regarding the comparable effectiveness of BFR-RE and traditional RE for increasing lower-limb 1RM should be interpreted cautiously. In this context, it should be noted that some previous meta-analyses have reported that BFR-RE in older adults is equally effective in increasing muscle mass but less effective in increasing muscle strength compared with traditional RE [7]. The lower effectiveness of BFR-RE may be related to a reduced neural response. Previous studies using conventional electromyography, including surface EMG, have shown that traditional resistance exercise elicits greater muscle activation [34–36]. Nevertheless, these findings should be interpreted with caution as surface EMG amplitude does not directly quantify motor unit recruitment and does not provide the same level of information as high-density electromyography. Cyclical motor unit recruitment, in which certain motor units temporarily stop firing to minimize muscle fatigue, is often not accounted for [37, 38]. Furthermore, because these studies were performed in young and healthy participants, their results may not be directly applicable to older adults [34, 35].
2. BFR-RE increases the isokinetic torque of knee flexors and extensors to a similar extent as traditional high intensity RE. However, the minimal clinically important difference for this outcome measure has not yet been established in the literature, and only one included study focused on this outcome. These findings suggest that BFR-RE may induce improvements in dynamic force production across different contraction modes, although the evidence is limited because isokinetic torque was assessed in only one study. Moreover, the lack of statistically significant between-group differences indicates that the observed changes should be interpreted as preliminary rather than conclusive evidence of comparable effectiveness.
3. BFR-RE increases the cross-sectional area of the *quadriceps femoris* muscle to a similar extent as traditional high intensity RE, as also confirmed by the meta-analysis [7]. One included study also reported an increase in the cross-sectional area of the hip adductor muscles and the *gluteus maximus* muscle [32]. However, this was a single study without a comparison group performing traditional RE, and therefore additional studies are required to draw firm conclusions. The observed increases in quadriceps cross-sectional area are clinically relevant because preservation of lower-limb muscle mass is important for maintaining mobility and independence in older adults. However, the finding that between-group differences were generally not statistically significant suggests that BFR-RE should be interpreted as a potentially comparable low-load alternative to high intensity RE, rather than as a superior hypertrophic stimulus. It should be noted that the increases in muscle mass observed with BFR-RE cannot be attributed solely to mechanical tension, which is considerably lower in BFR-RE. Other contributing factors may include metabolic stress [39], hypoxia [40], activation of mechanotransduction pathways [41], hormonal responses such as elevated growth hormone secretion [42], and intracellular swelling [43]. Importantly, most of these mechanisms have been studied primarily in younger individuals and without direct comparisons to traditional high intensity resistance exercise [34, 35]. Their relevance in older adults may also be influenced by age-related anabolic resistance, including impaired activation of mTORC1 signaling and a blunted muscle protein synthesis response after resistance exercise, which may attenuate hypertrophic adaptations and influence expected effect sizes [44]. Consequently, any conclusions regarding the relevance of these mechanisms for older adults remain tentative.
4. Functional tests were used in only one included study [32], without comparison to traditional high intensity RE. Consequently, no reliable conclusions can be drawn regarding the effect of BFR-RE on functional abilities in older adults. Additional studies incorporating appropriate functional tests (e.g., the Timed Up and Go test [45]) and comparison groups are required for a comprehensive understanding. The limited functional evidence is important because improvements in strength or muscle size do not necessarily translate directly into better performance in daily activities. Therefore, the absence of consistent functional outcomes limits the clinical interpretation of the findings, particularly for older adults whose primary goals are mobility, independence, and fall-risk reduction.

The results indicate that BFR-RE can improve both muscle strength and muscle mass of the lower limbs in older adults. These findings are also supported by previous research [46]. In all analysed studies, a positive effect of BFR-RE on lower-limb muscle strength was confirmed, supporting its potential as an effective alternative to high intensity RE, which is often less suitable for many older adults due to higher joint loading, comorbidities, and increased risk of injury.

Regarding the safety of BFR-RE, no major adverse side effects were reported in the included studies, which is encouraging but not sufficient to draw firm conclusions regarding safety. Nevertheless, this finding should be interpreted cautiously because the included studies had small sample sizes and included healthy older adults. Previous literature indicates that BFR exercise may induce acute increases in heart rate and blood pressure, with hemodynamic responses that may be particularly relevant for older adults with hypertension or cardiovascular disease [33, 47]. BFR may also increase perceived exertion, discomfort, and pain during exercise, which could affect tolerability and adherence, especially in individuals with chronic pain or musculoskeletal conditions [33, 48]. Therefore, careful screening, individualized cuff pressure prescription, and monitoring of cardiovascular and perceptual responses are recommended when applying BFR-RE in older adults [33, 48].

The results of our systematic review also reveal several limitations that should be considered when interpreting the findings. Among the main limitations are the pronounced heterogeneity of intervention protocols and measurement tools used, which further complicates direct comparison of results and limits the feasibility of future meta-analyses and quantitative synthesis. In all studies, sample sizes were relatively small [27–32], reducing the statistical power of the findings. In addition, none of the studies examined long-term effects, which limits understanding of the sustainability of BFR-RE effects. In several studies, muscle strength was expressed in kilograms; however, reporting joint torque (N⋅m) would be more appropriate [16]. Muscle force production cannot be directly compared between individuals without accounting for anatomical differences, such as limb length, which influence the resulting torque [49]. Reporting strength in kilograms may therefore be misleading, as two individuals with comparable muscle force but different limb lengths could produce different values. Some authors also pointed out potential effects of baseline differences between groups, such as differences in torque in one study [31], which may have influenced the final outcomes. Although all included studies were rated as at least methodologically acceptable, the risk of bias assessment using the RoB 2.0 tool (Table 2) identified several important limitations. None of the studies was judged to be at low risk of bias. Methodological concerns were most evident in domains related to the randomization process, deviations from intended interventions, and outcome measurement. These issues reduce the overall certainty of the available evidence. Another limitation is that this review focused on healthy older adults, while BFR-RE is often proposed as an alternative for individuals who cannot tolerate high intensity RE because of pain, comorbidities, or functional limitations. Therefore, the findings should not be directly generalized to older adults with conditions such as hypertension, diabetes, obesity, osteoporosis, osteoarthritis, or other chronic diseases. In addition, the definition of “healthy” was not always consistently reported or verified across the included studies. Some studies excluded only major, unstable, or uncontrolled conditions, meaning that participants with stable or common comorbidities may have been included. This limits the certainty with which the included population can be considered uniformly healthy and should be considered when interpreting the clinical applicability of the findings. A further methodological limitation is that study selection, data extraction, and risk of bias assessment were conducted using a verification approach rather than fully independent duplicate processes. Although all decisions were checked by two additional reviewers and disagreements were resolved by consensus, this approach may be more susceptible to anchoring bias than independent dual screening or extraction. However, the potential impact of this limitation was reduced by the small number of records remaining after title screening. The lack of prospective registration represents a methodological limitation, as it reduces transparency regarding potential deviations from the original protocol and may increase susceptibility to selective reporting bias. None of the included studies reported intention-to-treat analysis. Because dropout in exercise trials may be non-random and related to poorer responders, reliance on per-protocol analyses may have biased effect estimates upward. However, it should also be acknowledged that intention-to-treat analysis is often difficult to implement in small exercise trials without complete outcome data.

Despite these limitations, the results of this systematic review suggest that BFR-RE is a promising strategy for increasing lower-limb muscle strength and muscle mass in older adults. Due to the lower mechanical load, it is particularly suitable for individuals who are unable to perform high intensity RE. Future research should aim to improve methodological quality through clearly defined randomization procedures, standardization of measurement tools and intervention protocols, and blinding of participants and therapists. In addition, larger sample sizes should be included, other body regions should be examined, and long-term effects should be investigated to improve the quality of evidence and its applicability to clinical practice.

## Conclusions

This systematic review examined the effects of BFR-RE on muscle performance, muscle mass, and function in older adults. The findings indicate that BFR-RE effectively increases muscle strength and cross-sectional area of the lower-limb muscles, particularly the *quadriceps femoris* muscle, with effects often comparable to those of traditional high intensity RE. The low loads used in BFR-RE represent an important advantage for populations experiencing pain, comorbidities, or other functional limitations that make traditional RE difficult or impossible to perform.

The effects of BFR-RE on function in older adults remain insufficiently investigated. In addition, most of the included studies examined the effects of BFR-RE on the lower limbs, which limits the generalizability of the findings to the whole body. Meta-analytic findings suggest that BFR-RE is more effective than low intensity RE and has comparable effects on hypertrophy to high intensity RE, but a slightly smaller effect on strength [7]. The influence of cuff pressure prescription on BFR-RE outcomes remains unclear and requires further investigation.

Given the lack of studies including functional outcomes and upper-limb interventions, as well as the identified methodological limitations, future research is recommended with larger sample sizes, standardized intervention protocols, and the inclusion of four groups: BFR-RE, high intensity RE, low intensity RE, and a passive control group. Future studies should also investigate BFR-RE in older adults with common comorbidities, pain, and functional limitations to improve the translational relevance and clinical applicability of the findings. Such a design would enable a more robust evaluation of the effectiveness of different exercises approaches and contribute to the development of clinical guidelines.

Based on the current evidence, BFRE appears to be a promising low-load exercise strategy for improving lower-limb muscle strength and muscle mass in healthy older adults. However, this conclusion should be interpreted with caution due to the limited number of included studies, small sample sizes, heterogeneity of exercise protocols, and the fact that none of the included studies was judged to be at overall low risk of bias. Therefore, further high-quality randomized controlled trials are needed before firm clinical recommendations can be made.

## Data Availability

No datasets were generated or analysed during the current study.

## Appendix

**Table 3:** Characteristics of the included studies

AUTHOR (YEAR)	M/FX̄ AGE ± SD		INTERVENTION	X̄ PRESSURE, OCCLUSION DURATION, CUFF WIDTH	MEASUREMENT TOOLS		X̄ CHANGES AFTER INTERVENTION
Cook et al. (2017) [27]	5/776.5 ± 2.1		BFR-RE: 12 weeks, 2×/week, 3 sets, 60 s rest between sets and 180 s between exercises, 30 % 1RM LE and LF, 50 % 1RM HLP, to concentric failure, CON 3 s, ECC 3 s	1.5× brachial SBP (184 ± 25 mmHg), ≈ 5 min per exercise, rest between exercises WO, 5 cm	CSA-QF,1RM LE,1RM LF,1RM HLP,MVIC		↑ 1RM LE (9.1 kg),↑ 1RM HLP (18.7 kg),↑ CSA-QF (3.23 cm²)
	5/776.7 ± 2.68		HIRE: 12 weeks, 2×/week, 3 sets, 60 s rest between sets and 180 s between exercises, 70 % 1RM, LE, LF, HLP, to concentric failure, CON 3 s, ECC 3 s	n/a			↑ 1RM LE (21.2 kg)*,↑ 1RM LF (8.2 kg), ↑ 1RM HLP (31.7 kg), ↑ MVIC (19.3 N⋅m),↑ CSA-QF (2.86 cm²)
	5/774.8 ± 2.58		CG: 12 weeks, 2×/week, 3 repetitions of static upper body stretching + elbow flexion and extension and shoulder shrugs with free weights (≤ 2.27 kg) and elastic bands	n/a			n/a
Letieri et al. (2018) [28]	0/1168 ± 3.79		BFR-RE-H: 16 weeks, 3×/week, 3–4 sets, 30 s rest between sets and 90 s between exercises, 20–30 % 1RM, LE, LF, HLP, squats, 15 reps	80 % AOP (185.75 ± 5.45 mmHg), ≈ 3.89 min per exercise, rest between exercises WO, ? cm	IT-RE,IT-LE,IT-RF,IT-LF		↑ IT-RE (25.96 N⋅m), ↑ IT-LE (23.78 N⋅m), ↑ IT-RF (20.23 N⋅m), ↑ IT-LF (18.99 N⋅m)
	0/1169.4 ± 5.73		BFR-RE-L: 16 weeks, 3×/week, 3–4 sets, 30 s rest between sets and 90 s between exercises, 20–30 % 1RM, LE, LF, HLP, squats, 15 reps	80 % AOP (105.45 ± 6.5 mmHg), ≈ 3.89 min per exercise, rest between exercises WO, ? cm			↑ IT-LE (17.56 N⋅m), ↑ IT-RF (14.02 N⋅m), ↑ IT-LF (15.02 N⋅m)
	0/1066.75 ± 4.43		HIRE: 16 weeks, 3×/week, 3–4 sets, 60 s rest between sets and 90 s between exercises, 70–80 % 1RM, LE, LF, HLP, squats, 6–8 reps	n/a			↑ IT-RE (24.31 N⋅m), ↑ IT-LE (27.17 N⋅m), ↑ IT-RF (20.65 N⋅m), ↑ IT-LF (20.89 N⋅m)
	0/1271.27 ± 4.73		LIRE: ?	n/a			n/a
	0/1269 ± 6.39		PCG	n/a			n/a
Shimizu et al. (2016) [29]	16/472 ± 4		BFR-RE-L: 4 weeks, 3×/week, 3 sets, 30 s rest between sets and 60 s between exercises, 20 % 1RM, LE, HLP, BP, HR, 20 reps	Femoral artery SBP (163 ± 17 mmHg), entire exercise session, release during transition from LL to UL exercises, 10 cm		1RM LE,1RM HLP1RM BP1RM HR	↑ 1RM LE (8.9 kg),↑ 1RM HLP (15.7 kg)
	17/370 ± 4		LIRE: 4 weeks, 3×/week, 3 sets, 30 s rest between sets and 60 s between exercises, 20 % 1RM, LE, HLP, BP, HR, 20 reps	n/a			n/a
Vechin et al. (2015) [30]	M/F14/9	?/?; n = 865.0 ± 2	BFR-RE: 12 weeks, 2×/week, 4 sets, 60 s rest between sets, 20–30 % 1RM, HLP, 30/15/15/15 reps, CON 2 s, ECC 2 s	50 % SBP (71 ± 9 mmHg), entire exercise session, 18 cm		1RM HLP,CSA-QF	↑ 1RM HLP (99 kg; 54 %),↑ CSA-QF (4.2 cm^2^; 7.9 %)
		?/?; n = 862.0 ± 3	HIRE: 12 weeks, 2×/week, 4 sets, 60 s rest between sets, 70–80 % 1RM, HLP, 10 reps, CON 2 s, ECC 2 s	n/a			↑ CSA-QF (4 cm^2^; 6.6 %)
		?/?; n = 766.0 ± 5	PCG	n/a			n/a
Wang et al. (2023) [31]	11/1Me = 67IQR = 13		BFR-RE: 6 weeks, 3×/week, 4 sets, 30 s rest between sets, 30 % 1RM, LE, to concentric failure	50 % AOP + 5 mmHg (97 ± 3 mmHg), entire exercise session, 14 cm		MVIC,3RM LE,MET,6-MWT	↑ MVIC (11.5 %),↑ 3RM LE (15.6 %), ↑ MET (37 %),↑ 6-MWT (7.8 %)
	10/1Me = 67IQR = 13		PCG	n/a			n/a
Yasuda et al. (2014) [32]	3/671.3 ± 7.1		BFR-RE: 12 weeks, 2×/week, 4 sets, 30 s rest between sets and 90 s between exercises, 20–30 % 1RM, 30/20/15/10 reps, LE (CON 1 s, ECC 1 s), HLP (CON 1.3 s, ECC 1.3 s)	120 mmHg → each exercise session +10–20 mmHg = 270 mmHg, entire exercise session ≈ 11 min, 5 cm		CSA-QF,CSA-ADD,CSA-GM,1RM LE,1RM HLP,30sSTS	↑ CSA-QF (8.0 %), ↑ CSA-ADD (6.5 %), ↑ CSA-GM (4.4 %), ↑ 1RM LE (26.1 %), ↑ 1RM HLP (33.4 %), ↑ 30sSTS (18.3 %)
	2/867.7 ± 6		PCG	n/a			n/a

*1RM* one repetition maximum, *3RM *three repetitions maximum, *30sSTS *30-second sit-to-stand test, *6-MWT* 6-minute walk test, *AOP *arterial occlusion pressure, *BP *bench press, *BFR-RE* blood flow restriction resistance exercise, *cm *centimetre, *CON *concentric, *CSA-ADD *cross-sectional area of hip adductors, *CSA-GM* cross-sectional area of gluteus maximus, *CSA-QF* cross-sectional area of quadriceps femoris, *ECC* eccentric, *H* high, *HIRE *high intensity resistance exercise, *HLP* horizontal leg press, *HR* horizontal rowing, *IT-LF* isokinetic torque of left knee flexors, *IT-LE* isokinetic torque of left knee extensors, *IT-RF* isokinetic torque of right knee flexors, *IT-RE* isokinetic torque of right knee extensors, *IQR *interquartile range, *kg *kilogram, *LE *leg extension on machine, *LF *leg flexion on machine, *L *low, *LIRE *low intensity resistance exercise, *LL *lower limb, *Me *median, *MVIC *maximal voluntary isometric contraction, *MET *muscle endurance test, *mmHg *millimetres of mercury, *min *minutes, *N⋅m* newton-metre, *n/a* not applicable, *PCG *passive control group, *reps *repetitions, *s *seconds, *SBP *systolic blood pressure, *cm*² square centimetres, *SD *standard deviation, *UL *upper limb, *WO *without occlusion; X̄ = mean; ↑ = increase; * = compared with BFR-RE; / = not relevant; ? = not defined; ?/? = the sex distribution across the groups was not clearly reported

## Abbreviations

1RM: One repetition maximum
3RM: Three repetition maximum
AF: U Adequate follow-up
B. C.: Baseline comparability
BFR: Blood flow restriction
BFR-RE: Blood flow restriction resistance exercise
BFRE: Blood flow restriction exercise
BGSC: Between-group statistical comparisons
BOA: Blinding of assessors
BOS: Blinding of subjects
BOT: Blinding of therapists
C. A.: Concealed allocation
DFII: Deviations from intended interventions
EMG: Electromyography
ITTA: Intention-to-treat analysis
MOO: Measurement of outcomes
MSO: Microsoft office
N⋅m: Newton meter
OD: Outcome data
PEDro: Physiotherapy evidence database
PEAV: Point estimates and variability
PICOS: Population, intervention, comparison, outcomes, study design
PRISMA: Preferred reporting items for systematic reviews and meta-analyses
R. A.: Random allocation
RE: Resistance exercise
RoB 2.0: Risk of bias 2.0
SORO: Selection of reported outcomes
